# New Insights into Cerebral Vessel Disease Landscapes at Single-Cell Resolution: Pathogenetic and Therapeutic Perspectives

**DOI:** 10.3390/biomedicines10071693

**Published:** 2022-07-13

**Authors:** Megi Meneri, Sara Bonato, Delia Gagliardi, Giacomo P. Comi, Stefania Corti

**Affiliations:** 1 Dino Ferrari Centre, Department of Pathophysiology and Transplantation (DEPT), University of Milan, 20122 Milan, Italy; megi.meneri@unimi.it (M.M.); delia.gagliardi@unimi.it (D.G.); giacomo.comi@unimi.it (G.P.C.); 2 Neurology Unit, Fondazione IRCCS Ca’ Granda Ospedale Maggiore Policlinico, 20122 Milan, Italy; 3 Stroke Unit, Fondazione IRCCS Ca’ Granda Ospedale Maggiore Policlinico, 20122 Milan, Italy; sara.bonato@policlinico.mi.it; 4 Neuromuscular and Rare Diseases Unit, Department of Neuroscience, Fondazione IRCCS Ca’ Granda Ospedale Maggiore Policlinico, 20122 Milan, Italy

**Keywords:** cerebral vessel, atherosclerosis, cerebrovascular disease, transcriptomics, stroke, single-cell sequencing, single-cell omics, RNA

## Abstract

Cerebrovascular diseases are a leading cause of death and disability globally. The development of new therapeutic targets for cerebrovascular diseases (e.g., ischemic, and hemorrhagic stroke, vascular dementia) is limited by a lack of knowledge of the cellular and molecular biology of health and disease conditions and the factors that cause injury to cerebrovascular structures. Here, we describe the role of advances in omics technology, particularly RNA sequencing, in studying high-dimensional, multifaceted profiles of thousands of individual blood and vessel cells at single-cell resolution. This analysis enables the dissection of the heterogeneity of diseased cerebral vessels and their atherosclerotic plaques, including the microenvironment, cell evolutionary trajectory, and immune response pathway. In animal models, RNA sequencing permits the tracking of individual cells (including immunological, endothelial, and vascular smooth muscle cells) that compose atherosclerotic plaques and their alteration under experimental settings such as phenotypic transition. We describe how single-cell RNA transcriptomics in humans allows mapping to the molecular and cellular levels of atherosclerotic plaques in cerebral arteries, tracking individual lymphocytes and macrophages, and how these data can aid in identifying novel immune mechanisms that could be exploited as therapeutic targets for cerebrovascular diseases. Single-cell multi-omics approaches will likely provide the unprecedented resolution and depth of data needed to generate clinically relevant cellular and molecular signatures for the precise treatment of cerebrovascular diseases.

## 1. Introduction

Neurovascular diseases are a top cause of disability, morbidity, and death globally [1], with an estimated 143 million disability-adjusted life-years in 2019 expected to grow with an expanding and increasingly older population [2].

Therapeutic approaches in acute stroke are targeted at rapid recanalizing of the blocked artery, either with intravenous thrombolysis or with thrombus lysis by endovascular thrombectomy [3,4]. Often, this recanalization is not successful or is even inefficient in removing the occlusion. Although therapeutic strategies have significantly improved, this disease still poses an enormous burden on human health, and translational research in the cerebrovascular field is an urgent unmet need to promote healthy living worldwide.

Atherosclerosis is important in the onset and progression of cerebral vascular disease. Cells and fibrous and lipid-rich material can accumulate and form arterial plaques in mid-size and large arteries [5]. After the initial phases of plaque formation, leukocytes migrate into the site, promoting inflammation, and can trigger plaque growth, instability, erosion, or rupture. Plaque disruption causes platelet adhesion to the exposed vascular surface, activating the clotting cascade and leading to thrombosis, which can block the artery partially or completely and cause symptoms.

Genomic studies have highlighted potential genetic risk variants in cerebrovascular diseases, underscoring the relevance of identifying genetic factors associated with specific cell types. Dissecting the dynamic spectrum of a plaque’s cellular components is a challenge, but the resulting information may break new ground for therapeutic development.

Bulk RNA sequencing (RNA-seq) of rodent and human atherosclerotic plaques has yielded data on the cellular composition and main molecular pathways implicated in the plaque formation. Nevertheless, this technique does not offer cell-specific resolution and does not allow for identification of individual cells responsible for a signal, and the contributions of uncommon but significant cell subtypes can be lost in the noise of more common cellular populations.

Methods that could resolve this information at the single-cell level include single-cell RNA-seq and others under the single-cell omics umbrella, all of which can deepen and broaden results of pathological, imaging, and clinical investigations. Single-cell omics offers fundamental new tools for uncovering the mechanisms underlying atherosclerosis and cerebrovascular diseases. These techniques allow for the depiction of cellular phenotypes and their trajectory, including cellular plasticity and clonal expansion. Single-cell omics also can distinguish gene/protein expression signatures between healthy and disease conditions and various clinical phenotypes.

Single-cell methods likewise can aid understanding of the vascular modification process during stroke and other brain diseases. Here, as an example of the potential of these approaches, we review in detail the cellular landscape of atherosclerotic plaques, thrombi, and immune signaling as uncovered by single-cell omics investigations focusing on the potential this information holds to significantly influence treatment of human cerebral vessel disorders.

A search of the literature was performed in PubMed using key words searching and citation single cell sequencing and carotid atherosclerosis or single cell sequencing and cerebral vessel to identify articles published in English between 1 January 2000 and 25 June 2022.

## 2. Single-Cell Omics Technologies

With recent technological advancements, biological processes can be studied at the resolution of a single cell [6]. Single-cell multi-omics approaches include some key features: (1) single-cell isolation technologies, barcode labeling strategies, and sequencing to identify the diverse molecules that shape different phenotypes and states at the single-cell level, and (2) integrative bioinformatic analysis of omics data to characterize the molecular signatures of diverse cell populations to address the full-range complexity of their roles in pathophysiological events.

The most widely used technology is scRNA-seq [7], which captures gene expression in individual cells. Conventional bulk RNA-seq of cell populations gives the average gene expression signal from a group of cells in a sample and can distinguish effects of experimental conditions, but obscures individual cell signals. scRNA-seq gives the transcription profile of individual cells and can reveal phenotypic and functional differences between cells and even complex and rare cell populations. This technique makes it possible to track the evolutionary trajectory of specific cell lines during development. Single-cell technologies have advanced rapidly in recent years, enabling characterization of cellular heterogeneity in blood and tissue samples, including those associated with cardiac and cerebrovascular diseases [8,9,10,11,12].

The general workflow of scRNA-seq experiments usually includes: (1) isolation of single cells, i.e., by microfluidic platforms to separate cells; (2) cell lysis, mRNA capture, and reverse transcription into cDNA and its amplification; (3) preparation of barcoded cDNA libraries for next-generation sequencing (NGS); and (4) bioinformatic analysis. New types of cells and more clearly defined cellular subsets have been identified using cluster algorithms and unbiased dimension reduction. Single-cell RNA bioinformatic methods have been described in detail previously [13,14,15,16].

scRNA-seq makes it possible to divide cell populations into groups with different transcriptional features that confer on them characteristic metabolic states and biological functions [17,18,19,20]. Unbiased bioinformatic tools for analyzing scRNA-seq data are well suited for detecting novel, even rare, cell clusters (endothelial, vascular smooth muscle, and inflammatory cells) and predicting cell trajectories and dynamic biological processes [17]. With analysis of scRNA-seq datasets, researchers can study ligand–receptor relationships implicated in cell development, aging, and disease states. Furthermore, intercellular communications can be inferred in heterogeneous samples such as diseased cerebral vessels, atherosclerotic plaques, and thrombi [21,22,23]. The study of the cellular landscape of atherosclerosis often entails handling of limited clinical tissue samples such as artery biopsies, atherosclerotic plaques, or thrombi, making recovery of sufficient quantities of vital cells difficult. In particular, the cellular components of thrombi are very scarce, limiting RNA studies of mechanical thrombectomy [24]. RNA-seq methods for investigating small vessel disease, atherosclerosis, and thrombosis have been described in detail previously [22,25,26].

Other single-cell sequencing omics technologies can complement scRNA-seq, which does not capture cell–surface protein signatures. Cellular indexing of transcriptomes and epitopes by sequencing (CITE-seq) [27] can partially solve this problem, relying on oligonucleotide-barcode antibodies to translate protein information (antibody binding to cells) into a quantifiable signal that can be sequenced. Oligonucleotides linked to the antibodies can be transcribed and captured during scRNA-seq library preparation and sequenced, with the resulting information integrated into the rest of transcriptomic analysis.

Another approach that operates in tandem with scRNA-seq is the single-cell assay for transposase-accessible chromatin with sequencing (scATAC-seq) technique [28]. scATAC-seq builds sequence libraries by relying on hyperactive Tn5 transposase, which inserts adapters into open chromatin zones corresponding to transcribed DNA. The generated library is amenable to NGS and bioinformatic analysis of open chromatin and can uncover epigenetic changes caused by age, stress, environmental factors, disease, or chronic antigenic stimulation [29].

Methylation sequencing (methyl-seq) also can generate an epigenetic profile, depicting methylation in single cells [30]. Other technologies for generating epigenetic profiles are available [31].

Among the single-cell proteomic technologies, cytometry by time of flight (CyTOF) relies on mass spectroscopy to quantify metal isotope-labeled antibodies and other tags on individual cells. The basic methods for effectively analyzing complex CyTOF experiments have been described previously [32,33].

scRNA-seq techniques that isolate cells from a sample cannot capture local intercellular communication networks, and some spatial transcriptomic and proteomic methods have been developed to address this issue. These strategies integrate fluorescence in situ hybridization with transcription data or proteomics, permitting assessment of gene or protein expression in a single cell while showing cell location. Recent advances in spatial transcriptomics have enabled profiling of single cells [15]. Other examples of techniques for spatially identifying RNA molecules include methyl-seq, multiplex in situ hybridization, and in situ sequencing [34,35]. Spatial transcriptomics can capture distinct cell populations and gene expression state in specific zones of tissues and can inform cell–cell communication. Innovations in spatial transcriptomics methods will improve holistic understanding of cells involved in cerebrovascular diseases.

## 3. Single-Cell Sequencing in Preclinical Animal Models

Animal models are used to elucidate the link between blood flow and atherosclerosis lesion.

Indeed, disturbed flow alters gene expression in the vascular wall, leading to infiltration of leukocytes that can promote atherosclerosis. The process of transcriptome modification in different cell populations (endothelial, smooth muscle, macrophages, leukocytes) after alteration of blood flow is a main process in the pathogenesis. The most widely used in vivo platform to experimentally study flow perturbation in preclinical animal models is partial carotid ligation (PCL), which Kumar et al. have detailed in the mouse with a video explanation of endothelial cell collection for single-cell omics [36].

Andueza et al. [37] compared PCL and untreated mice and used scRNA-seq combined with scATAC-seq to analyze individual endothelial cells under perturbed or stable blood flow. Dataset analysis identified eight endothelial clusters from mice carotids, including inflammatory cells, fibroblasts, and smooth muscle cells. Trajectory studies demonstrated that artery endothelial cells are heterogeneous even with a stable flow. Endothelial cells are plastic, and disturbed flow profoundly changes endothelial phenotype, promoting cell transition from quiescent and atheroprotective phenotypes to subtypes with pro-atherogenic or pro-inflammatory features. Disturbed flow also promotes other phenotypic transitions, such as endothelial to mesenchymal, endothelial stem/progenitor cells, hematopoietic stem cells, and new immune cell-like phenotypes. The authors detected specific transcription factor–binding motifs (KLF4/KLF2) induced by stable flow and other factors (TEAD1, AP1, STAT1, RELA) induced by disturbed flow. They also found that blood flow modulates chromatin accessibility and transcription. These results show that abnormal blood flow influences expression at the transcriptional and epigenetic levels, promoting transdifferentiation of endothelial cells into proatherogenic cell phenotypes.

By reanalyzing these scRNA-seq and scATACseq datasets, the same group demonstrated a key role of kallikrein-related peptidase 10 (Klk10, a secreted serine protease) as a flow-regulated endothelial protein with positive anti-inflammatory, anti-atherogenic activities [38]. KLK10 expression is higher under stable flow but reduced under disturbed flow and protects against inflammatory signaling that counteracts the effect of disturbed flow. Administration of KLK10 in mice reduces arterial endothelial inflammatory processes associated with disturbed flow [38].

In an independent study, Li et al. [39] used a similar model of PCL in mice to generate and assess the impact of disturbed blood flow on vessels. scRNA-seq showed 15 separated cell clusters, including 10 relevant subpopulations related to disturbed blood flow. At least five distinct clusters were identified among the endothelial cells. Gene set variation analysis and single-cell trajectories identified the Dkk2hi endothelial cell subpopulation, which emerges after artery ligation, as a likely mechanosensitive cell cluster possibly generated from Klk8hi endothelial cells under disturbed flow. Of interest, genes expressed by Dkk2hi cells included Ngf, Col8a1, Kit, and Vcan, which previously have been described as associated with atherosclerosis. scRNA-seq showed three distinct disturbed flow-related macrophage clusters (Trem2hi, Res-like, and Birc5hi), highlighting a high macrophage diversity within the plaque.

Birc5 has been described as critical to macrophage function in atherosclerosis, namely, as a key element of macrophage apoptosis and recruitment. Disturbed flow promotes the epithelial-to-mesenchymal cell transition, and vascular smooth muscle cells exposed to disturbed flow express markers related to osteoblast differentiation that can contribute to arterial stiffness. Of other inflammation-related cell subpopulations, NK cells, CD4^+^ and CXCR6^+^ T cells, and granulocytes also have been detected under disturbed flow. Li et al. [39] generated a detailed single-cell atlas of arterial cells under disturbed blood flow, describing new cell subpopulations, patterns of gene expression in these populations, and how these events are related to atherosclerosis. Targeting these cellular and molecular processes may point in new therapeutic directions in atherosclerosis and cerebrovascular diseases.

Animal models are also useful for studying post-stenting carotid restenosis. Neointimal hyperplasia from vascular smooth vessel proliferation is the most important process in restenosis. Gao et al. [40] used single-cell sequencing of rat carotids, with the right carotid subjected to stenting injury and the left as unaffected control. In both data sets, they found 11 cell clusters grouped into six major cell types among which vascular smooth muscle cells were particularly abundant. Smooth muscle cells were altered into a variety of phenotypes that included a secretory phenotype correlated with hyperplasia after stenting. Data for disease risk factor genes yielded novel potential therapeutic targets such as Cyp7a1 and Cdk4, which are expressed by smooth muscle cells and fibroblasts and correlated with neointimal hyperplasia, an important target of future investigations.

### Single-Cell Sequencing in Humans

**Figure 1:**
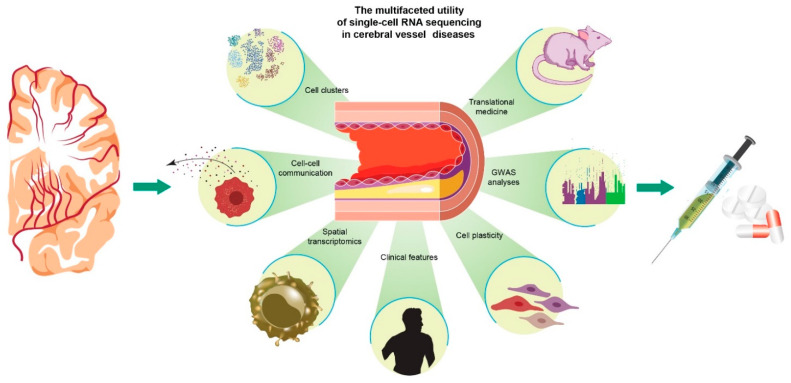
The multifaceted utility of single-cell RNA-seq in cerebral vessel diseases. scRNA-seq can be employed to unravel several features of cerebral vessel disease and the pathogenesis of atherosclerosis. This technology can reveal the cellular composition of a lesion, provide information on lineage tracing and cell evolutionary trajectory, and be applied to investigate cellular plasticity and clonality phenomena and match the expression contributions of individual cells to GWAS risk factors. Spatial transcriptomics might localize distinct cell populations in the vessels and plaques and elucidate their communication interplay. scRNA-seq can illuminate at the single-cell level the molecular features of different cerebrovascular disease clinical presentations and promote pathogenetic and therapeutic translational studies from animal models to patients.

Atherosclerosis and related cerebrovascular diseases develop with age and are linked to the accumulation of a spectrum of senescent cells and dysregulated inflammatory events. These conditions involve multifaceted players, including different vascular and metabolic components, age-associated chronic inflammatory processes within the blood circulation and at focal vascular walls, and the interplay between systemic and local sites. The highly diverse plaque cellular composition determines the clinical manifestations such as stroke. However, specific cell subpopulation phenotypes and status changes during atherogenesis (from initiation to a complex plaque) are still largely unknown. Single cell omics studies of human atherosclerotic lesions have demonstrated that T cells and macrophages are the most represented populations in atherosclerotic lesions [32,41,42]. Single-cell sequencing can highlight the complex cellular and molecular communication between immune cell subtypes and other vessel cells at initiation and progression of cerebrovascular disease. As noted, endothelial and smooth muscle cells are key plaque features, and single-cell sequencing is particularly apt for analysis of smooth muscle fates characterized by dynamic transdifferentiation processes, extracellular matrix production, and extensive intercellular molecular crosstalk. Dissecting these events can facilitate implementation of conceptually novel therapies for cerebrovascular diseases (Figure 1).

Most published scRNA-seq studies were conducted on tissues obtained during carotid endarterectomy (Table 1). In this surgery, the intima is collected together with the plaque, with the tunica media left in place. None of these studies were performed from material obtained during endovascular thrombectomy procedures, which may yield lower quality material with fewer cells because thrombi consist mainly of platelets and clotting factors, as bulk RNA-seq analysis has shown [24]. Careful tissue conservation from collection to analysis is crucial. The tissue must be dissociated before analysis, which erases spatial information and can introduce cell selection bias because some cell subsets can be lost during the digestion.

**Figure 2:**
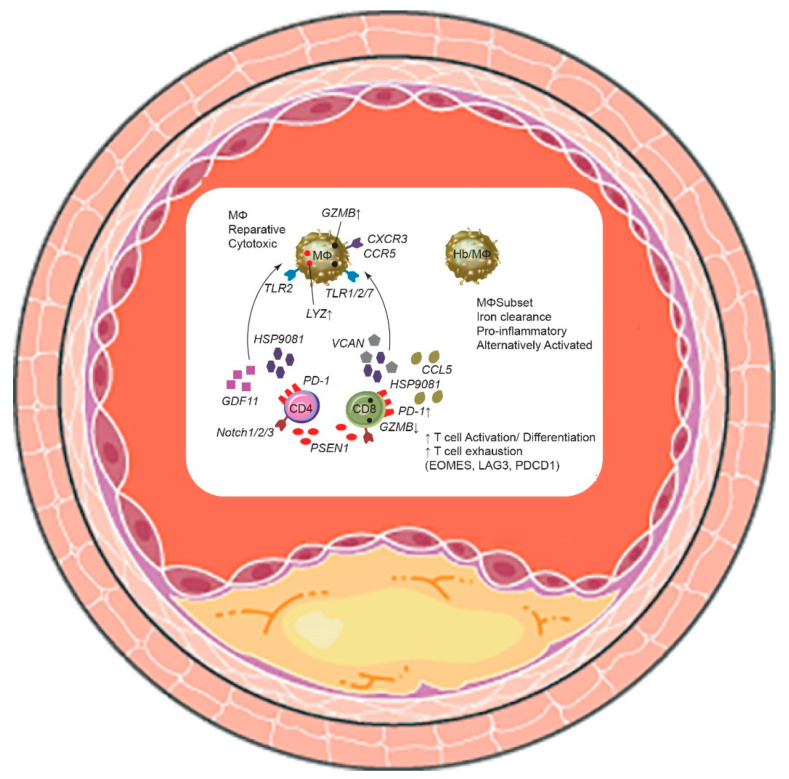
Immune cell–cell interactions of the human atherosclerotic plaque by single-cell omics. Main innate and adaptive immune cell to cell interactions in carotid plaques of symptomatic patients.

Fernandez et al. [22] provided a single-cell characterization of carotid atherosclerotic plaques from clinical samples by associating single-cell transcriptomic and proteomic analyses to develop a multimodal reference “map” of atherosclerosis in humans. Using scRNA-seq, CyTOF, and CITE-seq, they analyzed prospectively collected samples from 46 carotid endarterectomy patients. For this work, they profiled 7169 CD45^+^ cells from six atherosclerotic plaques using scRNA-seq and CITE-seq, and >90,000 CD45^+^ cells using CyTOF mass cytometry [22]. The most abundant plaque cell types were CD4^+^ and CD8^+^ T cells, and the most common in blood were CD8^+^ T cells, presenting considerable phenotypic heterogeneity. The phenotypes of the T cells and macrophages differed fundamentally between plaques from symptomatic patients (recent transient ischemic attack, stroke) and those from asymptomatic patients (no recent stroke). Distinct transcriptional changes in migration, activation, and differentiation of the cells accompanied the increased effector memory CD4^+^ T cell counts in plaques from symptomatic stroke patients. Furthermore, some T cell subgroups in these plaques showed RNA signatures compatible with T cell exhaustion (Figure 2). In contrast, T cells and macrophages in plaques from asymptomatic stroke patients displayed an activated phenotype and signs of pro-inflammatory interleukin (IL)-1ß and IL-6 signaling. Because IL-1 can be differentially expressed between asymptomatic and symptomatic patients, the response to anti-IL-1 therapies, as tested with canakinumab in the CANTOS study for atherosclerotic disease [43], might differ among patients depending on the IL-1 signaling pattern in the vessels.

The presence of exhausted and activated T cells in symptomatic patients within the same zone supports the hypothesis that over-activated T cells can transition to an exhausted phenotype, with progressive lack of T cell functions, likely promoted by chronic inflammation in the focal zone [8]. PD-1 is a master gene of T cell exhaustion and moderates the proatherogenic phenotypes of activated T cells in the artery wall of animal models, whereas its suppression exacerbates it. Thus, PD-1 inhibitors used in cancer treatment can activate T cells in plaques [22]. Fernandez et al. [22] showed a distinct expression profile of cells derived from symptomatic and asymptomatic patients and this study represents a first step in characterizing the mechanisms underlying the plaque diversity that leads to clinical events. A possible limitation of that work is its focus mainly on certain immunological-specific cell lineages and not the entire vascular wall. The findings of these studies point to the need for personalized treatment, including immune checkpoint-inhibiting drugs, to reach maximal therapeutic efficacy in cerebrovascular disease patients.

Cell–cell interaction studies have shown that T cell functional states are precisely governed by relationships with other T cells and macrophages. Infiltrating monocytes can transform into macrophages or dendritic cells, and a CyTOF study of immune cells has detected two subgroups of macrophages, M1 cells that are activated via classic pathways and the M2 phenotypes arising from alterative activation mechanisms [44,45]. Further transcriptional analysis has highlighted a variety of distinct macrophage populations with specific functions, including a subset with an activated phenotype, one that expresses proinflammatory genes, another that expresses genes involved in lipid metabolism similar to the functions of foam cells, and one that exhibits transcriptional signaling associated with anti-inflammatory processes. These results show that the spectrum of plaque macrophages is not accurately described with the classical oversimplified classification of M1/M2 subgroups [44,45].

An essential step will be determining if infiltrating lymphocytes and macrophages present features suggestive of a clonal origin. Clonal expansion of cells is based on a hypothesis derived from cancer research in which a parent cell divides and generates a progeny. This expansion can trigger pathological events in human diseases, including atherosclerosis, and some signs of T cell clonality in plaques have been detected from analysis of T cell receptors [22]. A new technique that combines scATAC-seq of mitochondrial RNA with scRNA-seq allows for clonal studies and lineage tracing in human samples [46]. Further scRNA studies will be needed to unravel the clonality of cells in the vasculature in response to atherosclerosis [47]. In this context, combining scRNA-seq with single-cell whole genome sequencing will be helpful for detecting somatic mutations that accumulate with aging and might underlie clonal expansion.

Depuydt et al. [23] examined human carotid atherosclerotic plaques, taking advantage of scRNA-seq and scATAC-seq to identify the related cells and analyze their transcriptome and epigenomic characteristics. Analysis of 3282 cells from 18 individuals using ScRNA-seq revealed 14 cell clusters, including 11 immunity-related clusters consisting of B cells, myeloid cells, mast cells, and T cells, and 3 non-immune cell clusters consisting of smooth muscle and endothelial cells. T lymphocytes comprised roughly 50% of plaque cells and myeloid cells 18.5%.

Subgroups of endothelial cells had pro-angiogenic characteristics and signs of phenotypic transdifferentiation, including endothelial-to-mesenchymal transition. CD4^+^ and CD8^+^ T lymphocytes presented various activation-state subtypes, including subclass phenotypes from cytotoxic to quiescent. Myeloid cells fell into two subgroups: proinflammatory macrophages characterized by IL-1B or TNF expression, and foam cell–like subgroups that were positive for a protein called triggering receptor expressed on myeloid cells 2, with a fibrosis-enhancing gene expression pattern.

ATACseq showed chromatin motif accessibility with transcription factor expression in myeloid cells that match the cytokines produced by T cells, suggesting intercellular communications between these cell groups. Finally, in silico analysis of deposited genome-wide association data revealed expression of cerebrovascular risk genes in plaque macrophages, endothelium, and smooth muscle cells as potential risk factors for cerebrovascular illness.

Five unique subsets of CD4^+^ T lymphocytes emerged with distinct developmental phases. Two clusters had a cytotoxic gene expression profile characterized by raised granzyme (GMZ) A (GMZA), GZMK, and perforin 1 (PRF1) levels and decreased GMZB levels, indicating the presence of cytotoxic CD4+ T cells in vasculature associated with disease. 

Open chromatin at the GZMB and GZMH loci validated the cytotoxic phenotype of CD4^+^ and CD8^+^ T cell clusters. Several genes encoding cytokines present an open chromatin pattern, supporting their active transcription. In particular, IFNG has open chromatin in two subtypes of T cells, the cytotoxic and effector T cells. IFNG can activate pro-inflammatory macrophages, and CD4/CD8 cells can be activated by dendritic cells that express IL12 (characterized by open chromatin in the enhancer). The coordinated IL12-IFNG signals may play a key role in activation of T cells in the plaque, with subsequent myeloid cell recruitment at the atherosclerotic sites. This pattern is consistent with other data showing the atherogenic action of IL12- IFNG in cerebrovascular disorders [48].

Depuydt et al. [23] also identified three populations of macrophages: two expressed proinflammatory phenotypes secreting TNF or IL-1ß, and the third population showing foam and profibrotic gene expression. This population also expressed proteins characteristic of smooth muscle cells, including alpha-actin, indicating that myeloid cells can acquire the vascular smooth muscle cell phenotype and that smooth muscle can transform into foam cells.

Among the smooth muscle cells, the authors identified two main subgroups of cells: one with a contractile phenotype and the other with a low level of typical muscle markers and a higher proportion of extracellular matrix proteins.

A study by Pan et al. [49] challenged this schematic view of a smooth muscle cell (SMC) phenotype in plaques. In comparing scRNA-seq profiles of human and mouse atherosclerotic lesions, they showed a broad spectrum of phenotypes generated via switching from a state into one or another. Smooth muscle cells dedifferentiated into an intermediate state during the atherosclerotic process in both mouse and human arteries. These cells were multipotent and could acquire other fates, including becoming macrophage-like or fibrochondrocyte-like, as well as re-differentiating into smooth muscle cells. Retinoic acid (RA) signaling was identified as a regulator of the dedifferentiated SMC and RA signaling was dysregulated in symptomatic human atherosclerosis. This phenotypic switching might represent a novel therapeutic target in atherosclerosis.

Li et al. [50] reanalyzed the data from [49], comparing the carotid artery plaques between patients who were symptomatic and asymptomatic. They found a remarkable diversity among endothelial cells, with three different subpopulations defined by specific features of antigen processing and antigen presentation, intercellular adhesion, or vascular smooth muscle cell hyperplasia. Of note, there was increased expression of genes linked to cell adhesion in asymptomatic plaques, and their up-regulation might represent a novel therapeutic target for increasing plaque stability and reducing stroke risk.

The utility of scRNA-seq for understanding how the smooth muscle cell phenotypic transition is generated and affects plaque stability also was confirmed in other work [51]. That study involved an extensive scRNA-seq of advanced human carotid endarterectomy samples, with analysis of 1287 single cells from 18 patients. These data were compared with scRNA-seq results from atherosclerotic lesions of mice subject to microdissection in which endothelial and smooth muscle cells were identified with lineage tracing. These results in turn were combined with those from bulk RNA-seq, chromatin immunoprecipitation sequencing, and dual lineage tracing in murine models. Alencar et al. [51] observed that Klf4 or Oct4 knockout in smooth muscle cells generated opposing genomic signatures, and their target genes regulated smooth muscle cell plasticity. In an earlier study, KLF4-dependent effects on the phenotype transition in vascular smooth muscle cells were pathogenic, and knockout of KLF-4 in an atherosclerosis mouse model diminished the size of the lesions [52]. A significant fraction of smooth muscle cells contributed to the Myh11^−^ and Lgals3^+^ population (two thirds of all smooth muscle cells express this marker), showing a gene expression pattern that was chondrocyte-like and diminished by Klf4 silencing. The activation of Lgals3 is pivotal to generating a population of cells that express genes characterized as stem cell markers and those associated with extracellular matrix remodeling. This subset gives rise to three other smooth muscle cell clusters, including Klf4-dependent osteogenic phenotypes that are coincident with plaque calcification and disruption. Overall, Klf4 pivoted transition of smooth muscle cells to a spectrum of phenotypes including Lgals3^+^ osteogenic cells that significantly contributed to late-stage atherosclerotic plaque pathogenesis. Whether calcification proves to be detrimental or protective in atherosclerotic plaques remains to be elucidated, as does the druggability of these transitions.

Alsaigh et al. [53] performed scRNA-seq of the total atherosclerotic core of plaques and their adjacent vessel portions from three people who underwent carotid endarterectomy. These authors profiled six principal cell populations (macrophages, endothelial cells, smooth muscle cells, NKT cells, and T and B lymphocytes) and relevant pathways of pathogenic events in endothelial cells and smooth muscle cells. Their findings demonstrated that the adjacent area is a continuum with respect to the morphological plaque. Adjacent cells collaborate in inflammatory processes in particular with endothelial cells secreting IL-6. TNFa pathways are active in both endothelial and smooth muscle cells in nearby areas. Indeed, several known pro-atherosclerotic genes are expressed. These data indicate that the pathological processes are not limited to the plaque but pervasively involve the entire vessel. The results illuminate an attractive opportunity to apply site-specific therapies to halt disease progression.

An essential point in the cerebrovascular diseases field is identifying relevant genetic risk factors that might represent possible therapeutic targets. Hundreds of candidate genetic variants for atherosclerotic disease and cardio/cerebrovascular risk factors have emerged in genome-wide association studies (GWAS), besides monogenetic risk factors [54]. Slenders et al. [42] applied single-cell gene expression profiling to atherosclerotic plaques considering cell-type–specific signatures and correlating these data with GWAS studies to unravel atherosclerosis-linked candidate gene–cell pairs. They projected GWAS loci onto single-cell transcriptomics data from diseased-associated samples, depicting both well-defined and new gene targets in association with their related cell populations. For this work, atherosclerotic lesion samples were obtained from 12 female and 26 male patients during a carotid endarterectomy surgery. The identified cell population clusters were similar to those reported in previous studies, in particular, overlap with that described by Pan et al. [46]. They then matched these expression data with GWAS-associated genes for different stroke subtypes (i.e., stroke and any ischemic stroke) and observed an overlap between genes expressed by the endothelial cell subpopulation and genomic risk factors. Among these genes are ESAM (endothelial cells adhesion molecules), LMNA, and SLC44A2. These results demonstrate that the genetic features of atherosclerosis do not arise from the immune system but rather from cells that constitute the arteries and control arterial wall integrity and plaque stability. This method can be employed for single-cell omics and diseases with complicated features.

Another example of a correlation of genomic and single-cell RNA-seq data has been published [55]. Polymorphisms at the HDAC9 locus are associated with the risk of carotid atherosclerosis and stroke. Chou and colleagues [55] performed single-nuclei RNA-seq analysis of clinical carotid tissue samples and showed that smooth vascular cells express HDAC9 in ways that might not only influence their phenotype but also increase macrophage homing and other inflammatory responses. These datasets relative to other published findings reveal a high proportion of smooth muscle vascular cells in the plaques. Of interest, this study showed a similar RNA signature of smooth muscle vascular cells with respect to [23], supporting the reproducibility of scRNA-seq approaches.

Recently, Winkler et al. [56] generated a human reference atlas of the single cells of normal and pathological cerebrovasculature. First, they profiled cells of the cerebral vessels from “normal” cerebral cortex from individuals who underwent lobectomies as a treatment for epilepsy. Large and small arteries and veins were isolated. scRNA-seq on 74,535 single cells from the vessels of five patients identified 15 major cell population clusters, including smooth muscle cells (MYH11^+^), pericytes (KCNJ8^+^), endothelial cells (CLDN5^+^), and perivascular fibroblasts (DCN^+^). Using marker genes defined from single-cell transcriptomes, they performed a spatial multiplexed transcriptomic analysis of different cerebrovascular cell states by combining multiplexed fluorescent in situ hybridization and immunostaining. Then, they applied multiplexed spatial transcriptomics to the tissues. This dataset highlighted the signatures responsible for arteriovenous phenotypic changes known as zonation. Besides endothelial cells, they also characterized the principal perivascular cellular subclasses in the brain, which are pericytes, smooth muscle cells, and perivascular fibroblasts. These findings confirm the presence of perivascular fibroblasts and of smooth muscle-like cells known as fibromyocytes in the human cerebrovasculature.

To apply this strategy in a practical context, these authors also applied scRNA-seq to cells isolated from arteriovenous malformation (AVM) samples. AVM is a relatively frequent cerebrovascular disease of arteriovenous patterning in which patients are prone to bleeding and stroke and represents a major cause of stroke in young people. Working with AVMs from the brains of five patients who underwent surgery for these lesions, the authors performed scRNA-seq on 106,853 cells from the dissected samples and identified 11 significant clusters. Spatial transcriptomic analysis characterized endothelial cells (CLDN5^+^), smooth muscle cells (TAGLN^+^), fibromyocytes (CCL19^+^), and perivascular fibroblasts (COL1A2^+^) in AVMs. A comparison of control and AVM data showed that endothelial cells in the AVMs presented mainly an arterial/venous phenotype without a transcriptional phenotype related to venules or capillaries, suggesting loss of normal zonation in the endothelial cells.

Distinct pathological transcriptomic states in the nidus were identified by scRNA-seq and spatial transcriptomics related to altered angiogenesis, cell communication, and immune cell crosstalk. Some molecular alterations have commonalities with undifferentiated endothelium in the embryo or angiogenic cells, whereas other gene expression profiles related to embryonic development and angiogenesis are remarkably absent or disrupted. The analyses indicated the presence of altered communication pathways comprising known AVM pathogenic pathways, such as the abnormal expression of mediators of angiopoietin, vascular endothelial growth factor, and transforming growth factor–ß, as well as new angiogenic and immune molecules in AVMs, including positivity for CD99, SPP1, and CALCR.

Hemorrhagic stroke is an extremely important consequence of AVMs, so characterizing the pathological cell states associated with AVM rupture is crucial. In this study, a spectrum of cellular and spatially heterogeneous immune reactions was observed in the AVMs. RNA analysis connected 871 differentially expressed genes to AVM rupture, many associated with vascular developmental pathways and inflammatory processes. The authors identified the cellular ontology of the cerebrovascular immune cells present in the AVM and detected the infiltration of distinct immune cell states. In particular, P2RY12^−^ monocytes, which are a subset of AIF1^+^ (IBA1) and were labeled as GPNMB^+^ Mo3, were highly present in ruptured AVMs and presented an activated phenotype.

The authors hypothesize that GPNMB^+^ monocytes contribute to vascular smooth muscle cell depletion, leading to AVM rupture and hemorrhage. These findings are expected to lead to new investigations in other cerebrovascular disorders to contribute to the mechanistic understanding and therapeutic development targeting the human cerebrovascular system.

## 4. Perspectives on the Application of Single-Cell Technology for Precision Medicine in Cerebrovascular Disorders

An atlas of human and experimental cerebrovascular diseases at the single-cell level would support precision neurology. Precise patient selection and stratification based on molecular markers are required to maximize therapeutic success. Data from single-cell omics reveal that vascular cells during atherosclerosis have increased tissue specialization, heterogeneity, and plasticity. By allowing analysis of cells from human samples that usually carry the limitation of size and quantity, single-cell omics enables the longitudinal characterization of the delicate cellular architecture of diseased human arteries.

Models of atherosclerosis in humans and mice differ fundamentally. The effects of high cholesterol, metabolic dysregulation, and environmental risk factors on different genetic backgrounds and aging are difficult to replicate in animal models. Animal models do not mimic human immune activity at plaques or spontaneous plaque instability [57]. Animal models can be used, however, for pharmacological screening and genetic engineering of the illness with cellular and molecular precision to explore the mechanistic influence of target genes and signaling pathways on plaque development and regression. Human sample analysis and in vivo animal studies thus are complementary.

scRNA-seq technology is clearly a robust and rapidly developing tool for generating expression profiles at the individual cell level and supporting the dissection of mechanisms of atherosclerosis, thrombosis, and tissue reaction in stroke. Indeed, single-cell experiments have revealed a more complex landscape of individual cell phenotypes, known and novel, than previously anticipated. In fact, the peculiarity of single-cell omics is its ability to capture heterogeneity and dynamic transformations in cell subpopulations. Cluster analysis has been used in the vast majority of the published data set, permitting the description of previously unknown cell types/states. Pseudo-time analysis has further contributed to the identification of relevant processes in atherosclerosis development. These analyses emphasize that there is some degree of transdifferentiation of a subset of cells into other phenotypes (such as smooth muscle cells into fibromyocytes), suggesting the presence of cell plasticity. The implementation of spatial gene expression information will expand the spectrum of possibilities for analysis, providing crucial new information on cell plasticity, spatio-temporal transcriptome profile, and intercellular communications in atherosclerotic lesions. In fact, while some degree of paracrine communication, i.e., through cytokines, is captured by conventional single-cell analysis, local ligand–receptor cell communications are largely lost.

A crucial aspect of single-cell omics data is the integration and comparative analysis of a dataset originating from different research groups, patients, batches, platforms, or species. The final objective is to describe in a reproducible way common cell phenotypes detected across various scRNA-seq datasets by ensuring that clustering is robust and consistent and dimensionality reduced among studies. The generation of a consensus nomenclature of cell types will allow comparison of the results obtained from different groups and facilitate the evaluation of common and divergent properties of cell types across species and thus, translational studies from animals to humans.

## 5. Conclusions

Cost reductions for single-cell sequencing experiments will be helpful in opening the way to larger sample sizes and increased power in studies as well as in facilitating the association of other technologies with single-cell sequencing. A large majority of scRNA-seq datasets are available in public databases for independent analysis or analysis using online tools such as PlaqView [58] (http://plaqview.uvadcos.io/, accessed 25 May 2022). Overall, the volume and complexity of data that will be generated will provide a challenge in the framework of big data that might be solved with artificial intelligence applications coupled with user friendly interfaces. Single-cell transcriptomics offers new opportunities for unraveling the intricacy of cerebrovascular illness, and analysis of cerebral artery disease, atherosclerotic tissue, and thrombi can be facilitated by single-cell technology. Recent studies have produced a reference map of immunological abnormalities associated with atherosclerotic plaques in human carotid arteries, which may help in development of immunomodulation therapies, such as immune checkpoint regulation. Targeting inflammation to halt or reverse cerebrovascular disease [59] has been proposed recently, but single-cell resolution data suggest that more fine-tuning of the treatment is needed to achieve an adequate response.

Individualized treatment must be linked to underlying plaque heterogeneity and specific mechanisms of disease development. scRNA-seq could generate precise diagnostic tools and biomarkers for risk assessment and individualized therapy. Single-cell omics may facilitate understanding of the implications of cerebrovascular risk variables such as smoking, age, gender, or sex, and diabetes for disease onset and progression, as well as regression of plaques with lipid-lowering drugs, and the molecular and cellular events that influence cerebrovascular phenotypes and outcomes. Combining plaque single-cell omics data with CT/MRI/scan or doppler imaging data will maximize clinical value.

Standardized cross-study methods can be used to assess atherosclerosis and thrombosis in humans to identify relevant preclinical platforms that can validate recognized targets and related pathways. These methods also can be used to develop new candidate therapies to propose for clinical studies of cerebrovascular diseases.

## Figures and Tables

**Table 1:** Single cell RNA seq analysis in human cerebral vessels and atherosclerotic plaques.

Data ID	Population	Cells	The Focus of the Paper	Findings
Fernandez_2019 Carotid Artery Plaque Endarterectomy	6 patients	7.169 CD45^+^ cells	Immune cells in the plaques	T cell exhaustion and different IL-1 signaling patterns in symptomatic patients
Depuit_2020 Carotid Artery Plaque Endarterectomy	18 patients	3.282	All the cells in the plaques	IL12-IFNγ axis, an important feature of T-cell activation in the plaque
Pan_2020 Carotid Artery Plaque Endarterectomy	3 patients	8.867	Vascular smooth muscle cell	Retinoic acid signaling modulates SMC and atherosclerosis progression
Alencar_2020 Carotid Artery Plaque Endarterectomy	18 patients	1.287	Vascular smooth muscle cells	SMC phenotypic is regulated by Klf4 and Oct4
Alsaigh_2020 Carotid Artery Plaque Endarterectomy + Adjacent	3 patients	51.981	All the cells in the plaques	TNFa pathways are active in both endothelial and SMC
Slenders_2021 Carotid Artery Plaque Endarterectomy	38 patients	5.633	Genetic risk factors	New gene target in cerebrovascular disease: ESAM LMNA, SLC44A2.
Chou_2021 Carotid Artery Plaque Endarterectomy	7 patients	6.049	Vascular smooth muscle cells	HDAC9 modifies VSMC phenotype and immune cell recruitment in carotid disease
Winkler_2022 Large and small arteries and veins Epileptic patients that underwent lobectomy	5 patients	74.535	Normal human brain vasculature.	Cellular and molecular profiles of the adult human cerebrovasculature
Winkler_2022 arteriovenous malformation (AVM)	5 patients	106.853	Malformed human brain vasculature	AVM rupture is leaded by GPNMB+ monocytes

## Data Availability

Data sharing not applicable.
